# Effect of altering breathing frequency on maximum voluntary ventilation in healthy adults

**DOI:** 10.1186/s12890-018-0650-4

**Published:** 2018-05-24

**Authors:** Eric V. Neufeld, Brett A. Dolezal, William Speier, Christopher B. Cooper

**Affiliations:** 1 Exercise Physiology Research Laboratory, Departments of Medicine and Physiology, David Geffen School of Medicine at UCLA, 10833 Le Conte Avenue, 37-131 CHS Building, Los Angeles, CA 90095 USA; 2 Medical Imaging Informatics, Department of Radiology, David Geffen School of Medicine at UCLA, Los Angeles, CA USA

**Keywords:** Pulmonary function test, Standardization, Breathing frequency, Exercise testing, Ventilatory reserve, Maximal exercise ventilation, Forced expiratory volume in 1 s

## Abstract

**Background:**

Compared to other pulmonary function tests, there is a lack of standardization regarding how a maximum voluntary ventilation (MVV) maneuver is performed. Specifically, little is known about the variation in breathing frequency (*f*_R_) and its potential impact on the accuracy of test results. This study examines the effect of several preselected values for *f*_R_ and one self-selected *f*_R_ (*f*_Rself_) on MVV.

**Methods:**

Ten participants performed MVV maneuvers at various *f*_R_ values, ranging from 50 to 130 breaths·min^− 1^ in 10 breaths·min^− 1^ intervals and at one *f*_Rself_. Three identical trials with 2-min rest periods were conducted at each *f*_R_, and the sequence in which *f*_R_ was tested was randomized. Ventilation and related parameters were measured directly by gas exchange analysis via a metabolic measurement system.

**Results:**

A third-order polynomial regression analysis showed that MVV = − 0.0001(*f*_R_)^3^ + 0.0258(*f*_R_)^2^–1.38(*f*_R_) + 96.9 at preselected *f*_R_ and increased up to approximately 100 breaths·min^− 1^ (r^2^ = 0.982, *P* < 0.001). Paired *t*-tests indicated that average MVV values obtained at all preselected *f*_R_ values, but not *f*_Rself_, were significantly lower than the average maximum value across all participants. A linear regression analysis revealed that tidal volume (V_T_) = − 2.63(MVV) + 300.4 at preselected *f*_R_ (r^2^ = 0.846, *P* < 0.001); however, this inverse relationship between V_T_ and MVV did not remain true for the self-selected *f*_R_. The V_T_ obtained at this *f*_R_ (90.9 ± 19.1% of maximum) was significantly greater than the V_T_ associated with the most similar MVV value (at a preselected *f*_R_ of 100 breaths·min^− 1^, 62.0 ± 10.4% of maximum; 95% confidence interval of difference: (17.5, 40.4%), *P* < 0.001).

**Conclusions:**

This study demonstrates the shortcomings of the current lack of standardization in MVV testing and establishes data-driven recommendations for optimal *f*_R_. The true MVV was obtained with a self-selected *f*_R_ (mean ± SD: 69.9 ± 22.3 breaths·min^− 1^) or within a preselected *f*_R_ range of 110–120 breaths·min^− 1^. Until a comprehensive reference equation is established, it is advised that MVV be measured directly using these guidelines. If an individual is unable to perform or performs the maneuver poorly at a self-selected *f*_R_, ventilating within a mandated *f*_R_ range of 110–120 breaths·min^− 1^ may also be acceptable.

## Background

Formerly referred to as maximum breathing capacity, maximum voluntary ventilation (MVV) is a pulmonary function test (PFT) that measures the maximum amount of air a person can inhale and then exhale with voluntary effort. The test is measured in liters per minute (L·min^− 1^), but data is only collected for 12–15 s and then extrapolated to 1 m in order avoid prolonged hyperventilation by the participant. While the test has been used less over the recent decades due to its fewer applications than the forced expiratory volume in 1 s (FEV_1_), MVV still possesses clinical utility. Performing the maneuver is contingent on several factors, including respiratory system mechanics (obstructive or restrictive) and ventilatory muscle endurance. Therefore, the test provides a broad assessment of respiratory system function [1]. Abnormal MVV results are valuable in evaluating various neuromuscular disorders [2–4] and predicting the risk for postoperative complications [1, 5]. MVV also remains useful in cardiopulmonary exercise testing as a measure of ventilatory capacity, especially in determining an individual’s ventilatory reserve—the difference between MVV and maximal exercise ventilation (${\dot{V}}_{Emax}$)—which aids in the diagnosis and differentiation of pulmonary and cardiovascular diseases [6–10].

What primarily distinguishes MVV from other PFTs, however, is the lack of standardization regarding how the maneuver is performed. A major component of the test is breathing frequency (*f*_R_), but little consensus exists on precisely what *f*_R_ or range of *f*_R_ values yield optimal results. The majority of both past and current research that employed MVV does not provide the frequency at which participants ventilated as part of the methodology [11–13]. Moreover, the few studies that do provide this information offer diverging suggestions. One of the earliest investigations on this matter concluded that ventilating at 70 breaths·min^− 1^ maximized MVV [14]. A later study utilized an *f*_R_ range of 60–120 breaths·min^− 1^ [15] while another argued against accepting results obtained with a frequency of less than 65 breaths·min^− 1^ [16]. A third investigation suggested a narrower window of 70–110 breaths·min^− 1^ [17]. The American Thoracic Society/European Respiratory Society (ATS/ERS) Task Force offered perhaps the most widely accepted range of 90–110 breaths·min^− 1^ with an ideal rate of approximately 90 breaths·min^− 1^, but also declared that no specific *f*_R_ can be mandated due to a lack of research on the topic [18].

The limited evidence supporting an ideal *f*_R_ range is problematic. Considering both the diagnostic and prognostic value of MVV, as well as its ability to assess participant compliance during pulmonary function testing [19], inaccurate results due to poor standardization may have substantial clinical implications. Consequently, it is imperative that the variety of current guidelines be evaluated. The aim of this investigation, therefore, was to determine whether there is an optimal *f*_R_ at which the MVV maneuver ought to be performed, i.e., to consistently provide the highest outcome value. This was accomplished by conducting MVV tests at a wide range of *f*_R_ utilizing a repeated-measures design. In addition to examining preselected *f*_R_, this study also explored the effect of a self-selected *f*_R_ (*f*_Rself_). Given that synchronizing breathing frequency to a rhythm-keeping device at a preset rate may feel unnatural or interfere with performance, we hypothesized that participants would maximize MVV by ventilating at a *f*_R_ uniquely self-selected by each individual rather than breathing within a predetermined range.

## Methods

### Study design

Ten healthy, non-smoking adults (six men) were recruited from the University of California, Los Angeles (UCLA) community to participate in this study. Nine were undergraduate students aged 18–22 years old and one was a 46 year-old university employee (height 1.72 ± 0.13 m; mass 67.1 ± 16.3 kg). The UCLA Institutional Review Board approved this study, and all participants provided informed consent prior to enrollment.

Each participant performed MVV maneuvers using nine different preselected *f*_R_s ranging from 50 to 130 breaths·min^− 1^ in increments of 10 breaths·min^− 1^ as measured by a metronome. Additionally, all participants performed a maneuver using *f*_Rself_. In this condition, subjects performed the test without the aid of a metronome, and were instead instructed to breathe as rapidly and deeply as possible without inducing significant discomfort. The resulting frequency was then recorded. For each frequency, including *f*_Rself_, participants performed three trials for a total of 30 tests (10 per day with 2-min rest between each) over three consecutive days to allow for adequate recovery. To limit order and practice effects, a random number generator determined the sequence in which *f*_R_ was tested for each participant.

### Data acquisition

All data were obtained using a metabolic measurement system (Oxycon Pro™; CareFusion, Yorba Linda, CA) that underwent volume and gas composition calibrations prior to each testing session. All tests occurred in a laboratory setting where the ambient temperature and humidity were measured and entered into the system before calibration. The volume calibration was done mechanically with known volumes of air, while the composition calibration was performed using ambient air and a gas tank of 16% O2, 4% CO2 to encompass the range found in normal exhaled air. Participants were seated upright in a stationary chair, wore a nose clip, and breathed through a mouthpiece that connected to the metabolic cart. Participants were also instructed to refrain from eating or engaging in vigorous exercise at least before testing.

Prior to performing the first MVV maneuver, slow vital capacity (SVC) was measured using the same system. Participants were instructed to inhale as much air as possible in a single breath and then exhale, completely emptying the lungs. This value was obtained to compare the ratio of the tidal volume (V_T_) during MVV to SVC. In addition, participants took normal breaths to establish a baseline respiratory exchange ratio, which was used as a metabolic indicator to determine when a participant had achieved sufficient rest between trials. Once this value had been established, MVV testing began. For each trial, participants were instructed to maximize ventilation by inhaling and exhaling as deeply as possible for 12 s. As frequent vigorous respiration can cause dry mouth, water was provided if requested between tests. During trials defined by a preselected *f*_R_, participants listened to a corresponding preset tempo from a metronome and timed their breaths accordingly. In between each beat, both an inhalation and an exhalation were performed. During the trials associated with *f*_Rself_, no metronome was used— participants freely maximized breathing while the metabolic cart recorded *f*_R_, the value of which was blinded until after a maneuver was completed. In addition to *f*_R_ and MVV, which were recorded and analyzed for all trials, breath-by-breath measurements of V_T_ and partial pressure of end-tidal CO_2_ (PETCO_2_) were also obtained for all maneuvers except those at the preselected *f*_R_ of 130 breaths·min^− 1^ (*f*_R130_) (Table 1).

**Table 1:** Outcome variables (scaled and absolute values)

*f*_R_	MVV		V_T_		P_ET_CO_2_
(breaths·min^− 1^)	(%)	(L·min^− 1^)	(%)	(L)	(mmHg)
50	76.2 ± 9.9^*†^	125.1 ± 45.3	99.1 ± 1.6	2.4 ± 0.9	15.3 ± 2.0
60	81.7 ± 13.9^†^	131.4 ± 52.0	88.4 ± 9.0^†^	2.2 ± 0.8	15.0 ± 3.3
70	84.3 ± 11.9^†^	134.1 ± 47.2	78.5 ± 5.4^†^	1.9 ± 0.7	14.5 ± 2.9
80	87.5 ± 8.3^†^	139.4 ± 50.2	72.1 ± 12.3^*†^	1.7 ± 0.6	14.2 ± 2.7
90	91.4 ± 5.9^†^	144.1 ± 49.6	67.7 ± 11.5^*†^	1.6 ± 0.5	13.3 ± 2.1
100	93.3 ± 3.7^†^	147.6 ± 51.5	62.0 ± 10.4^*†^	1.5 ± 0.5^†^	14.2 ± 2.7
110	92.2 ± 9.5^†^	144.9 ± 48.4	51.9 ± 19.9^*†^	1.3 ± 0.6	13.6 ± 2.2
120	90.2 ± 10.2^†^	144.7 ± 58.3	50.5 ± 10.3^*†^	1.2 ± 0.5	13.0 ± 2.4
130	81.0 ± 11.4^†^	129.6 ± 52.1	n/a^b^	n/a^b^	n/a^b^
Self-Selected (69.9 ± 22.3)	91.1 ± 16.5	144.8 ± 59.4	90.9 ± 19.1	2.1 ± 0.7	13.7 ± 2.0
Maximum^a^	–	157.7 ± 52.5	–	2.4 ± 0.9	17.1 ± 2.5

Values are presented as mean ± SD both 1) scaled as a percentage of the maximum and 2) absolute. Statistical analysis was performed only on scaled values

All values are reported as the percent of the maximum value obtained using preselected *f*_R_ values

*Abbreviations:* *f*_*R*_ breathing frequency*, MVV* maximum voluntary ventilation*, V*_*T*_ tidal volume*, PETCO*_*2*_ partial pressure of end-tidal CO2

*P < 0.05 when compared to self-selected *f*_R_

†*P* < 0.05 when compared to maximum

^a^Highest value obtained across all *f*_R_ values for every participant

^b^Values were missing from data collection

### Statistical analysis

Data for each subject were scaled relative to the maximum value recorded during preselected *f*_R_ and reported as the mean ± standard deviation (SD) for all variables. Sample size was estimated from a combination of pilot testing and preliminary power calculations based on an alpha level of 0.05 and a beta level of 0.20 [20]. As suggested by the ATS/ERS Task Force [18], only the test that resulted in the highest MVV out of the three trials per *f*_R_ was used for data analysis. The overall effect of *f*_R_ was tested using repeated-measures analysis of variance (ANOVA); comparisons were made between the outcome value at every *f*_R_ and the maximum value using paired *t*-tests. The average MVV values were found across subjects for each *f*_R_, and the relationship between these values and the corresponding *f*_R_ and V_T_ were analyzed using polynomial regression. The statistics were calculated in MATLAB (version 8.6.0; MathWorks, Inc., Natick, MA) and significance was determined using an alpha level of 0.05.

## Results

**Fig. 1:**
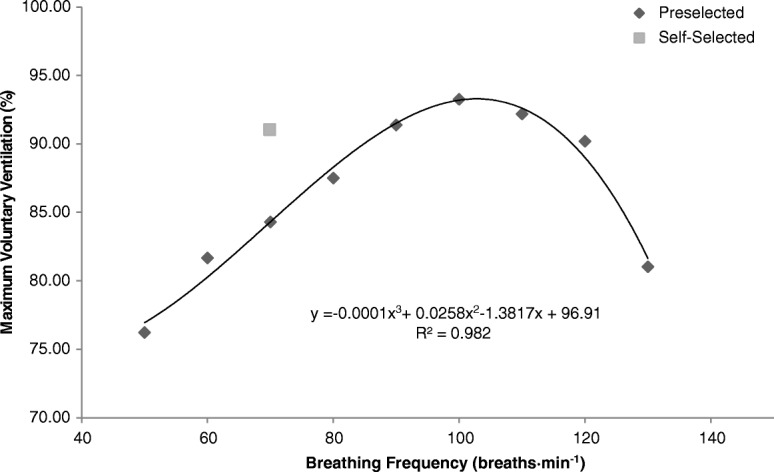
Relationship between the average maximum voluntary ventilation (MVV) across subjects and average breathing frequency (*f*R). MVV values are presented as a percentage (%) of the maximum. A polynomial regression analysis of the preselected *f*Rs yielded a third-order relationship (R^2^ = 0.982, *P* < 0.001)

All ten participants successfully performed three trials at all ten *f*_R_ values. As demonstrated in Fig. 1, a third-order polynomial regression analysis showed that MVV = − 0.0001(*f*_R_)^3^ + 0.0258(*f*_R_)^2^–1.38(*f*_R_) + 96.9 at preselected *f*_R_ and increased up to approximately 100 breaths·min^− 1^ (r^2^ = 0.982, *P* < 0.001). A paired *t-*test revealed that the MVV value obtained at an average *f*_Rself_ of 69.9 ± 22.3 breaths·min^− 1^ (91.1 ± 16.5% of maximum) was not significantly different from the value measured at the roughly equivalent preselected *f*_R70_ (84.3 ± 11.9% of maximum; 95% confidence interval of difference: (− 2.7, 16.3%), *P* = 0.190). When the MVV values at all *f*_R_ values were compared to the average maximum value for all subjects, a repeated-measures ANOVA showed a significant effect of *f*_R_ on MVV (*P* < 0.001). Further statistical analysis showed that results obtained at every preselected *f*_R_ were significantly lower than this maximum value, but those measured at *f*_Rself_ were not (Table 1). If multiple comparisons are controlled for using a Bonferroni correction, all preselected *f*_R_ values other than *f*_R110_ and *f*_R120_ remain significantly different from the maximum.

**Fig. 2:**
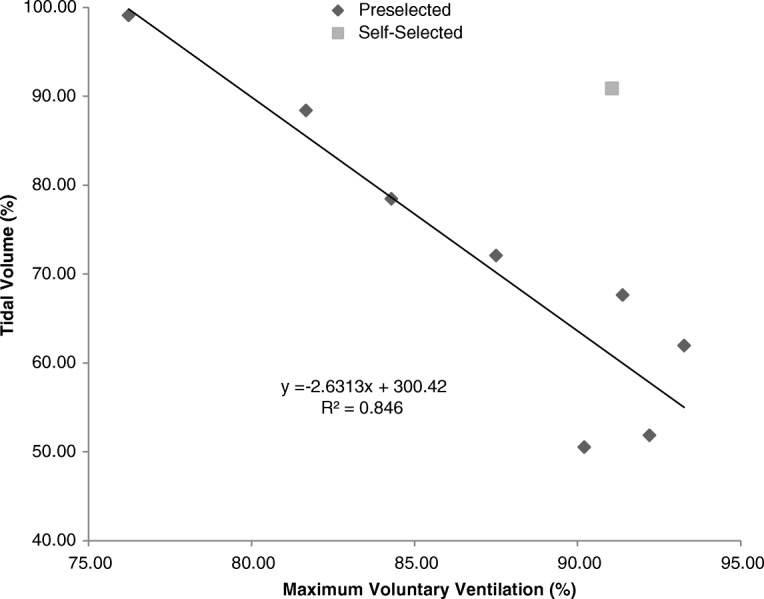
Relationship between the average tidal volume (V_T_) across subjects and the average maximum voluntary ventilation (MVV). V_T_ and MVV values are presented as a percentage (%) of their respective maxima. A linear regression analysis revealed a significant decreasing trend for the preselected breathing frequencies (R^2^ = 0.846, *P* < 0.001)

As demonstrated in Fig. 2, a linear regression analysis revealed that V_T_ = − 2.63(MVV) + 300.4 at preselected *f*_R_ (r^2^ = 0.846, *P* < 0.001). This steadily decreasing trend in V_T_ was observed as the preselected *f*_R_ and MVV increased, but paired *t-*tests showed that this inverse relationship did not remain true for *f*_Rself_. The V_T_ obtained at this *f*_R_ (90.9 ± 19.1% of maximum) was significantly greater than the V_T_ associated with the most similar MVV value (at *f*_R100_, 62.0 ± 10.4% of maximum; 95% confidence interval of difference: (17.5, 40.4%), *P* < 0.001). By contrast, none of the PETCO_2_ data measured at the preselected *f*_R_ differed significantly from that of *f*_Rself_, and all were significantly less than the average PETCO_2_ maximum.

## Discussion

Our study, to the best of our knowledge, is the first that examines the effect of altering *f*_R_ on MVV. After testing preselected *f*_R_ values ranging from 50 to 130 breaths·min^− 1^ as dictated by a metronome and one self-selected *f*_R_ by each participant, we found that trials conducted at 90–120 breaths·min^− 1^, and the self-selected rate were equally successful at maximizing MVV. This result differed from our hypothesis where we predicted that only *f*_Rself_ would yield the highest MVV. When compared to the aforementioned guidelines for optimizing MVV of 60–120, 70–110, and 90–110 breaths·min^− 1^ suggested by Dillard et al. (1993) [15], Morris (1976) [17], and the ATS/ERS Task Force [18] respectively, our findings disagreed with the first and second recommendations but closely resembled the third. However, our data do not support the ATS/ERS Task Force’s recommended goal of 90 breaths·min^− 1^. Considering that participants maximized MVV by breathing at *f*_R90–120_ and *f*_Rself_, we recommend choosing either of these approaches rather than aiming for a single value of *f*_R_. Furthermore, because maximal values were obtained at *f*_Rself_, our data suggest that no rhythm-keeping instrument or preselected *f*_R_ range may be necessary at all.

Interestingly, while the trials defined by a *f*_R70_ yielded a significantly lower MVV than the mean maximum for all subjects, they were not statistically different than the MVV value obtained at *f*_Rself_ (69.9 ± 22.3 breaths·min^− 1^, nearly equivalent to 70 breaths·min^− 1^). This is likely explained by the variance, which suggests that while breathing at a self-selected rate may increase the likelihood of achieving one’s true MVV, this chosen rate may not be equal among all individuals. Another noteworthy observation stemmed from the differences in V_T_ measured at the preselected and self-selected *f*_R_. When plotted against MVV, V_T_ steadily decreased at the preselected *f*_R_ values. This was unsurprising considering that MVV values were greater at higher *f*_R_ and as *f*_R_ increases, the tidal volume may decrease in order to sustain the faster rate. However, it is also possible that during exercise, increased intra-thoracic pressure and work of breathing can permit concomitant increases in *f*_R_ and V_T_. Interestingly, the data showed that the V_T_ associated with the MVV value obtained at *f*_Rself_ deviated from the trend observed at the preselected *f*_R_ values—it was significantly greater than its most similar preselected counterpart (obtained at *f*_R100_). This suggests that when breathing at a self-selected rate, individuals are more likely to utilize an optimal combination of slower deep breaths and faster shallower breaths to maximize MVV. Additionally, matching the rate of one’s breathing to a metronome or other rhythm-keeping instrument can feel distracting and unnatural to the participant, especially compared to ventilating at a natural rate during an assessment of ${\dot{V}}_{Emax}$, which may decrease the likelihood of an accurate measurement [1, 21].

Unlike MVV and V_T_, there were no significant relationships observed between *f*_R_ and PETCO_2_. This is somewhat unexpected as PETCO_2_ is known to decrease as the ventilation increases. The lack of this trend in the data may be partially explained by the missing values associated with *f*_R130_. It also is plausible that the inverse relationship between *f*_R_ and PETCO_2_ is not evident until the rate of ventilation is much greater than what was tested in this study. Moreover, it is important to note that the missing values for V_T_ at *f*_R130_ may not have fit the strong, negative linear correlation demonstrated in Fig. 2. Due to the sharp decrease in MVV from *f*_R120_ to *f*_R130_, it is unlikely that the corresponding reduction in V_T_ would have been of the same magnitude; however, it is also improbable that this single data pair would have rendered the aforementioned relationship between V_T_ and MVV non-significant.

Regarded as one of the most long-standing PFTs, equations developed to predict MVV from more widely applicable parameters, such as FEV, have existed since the mid-twentieth century. The most common and simplistic of these include MVV = FEV_1_ × 35 [22]; FEV_1_ × 37.5 [23]; and FEV_1_ × 40 [24]. And while some still prefer to utilize these equations over a direct assessment, substantial evidence has highlighted the limitations of such practice. These along with many similar reference equations fail to account for a number of physical characteristics that have been shown to influence MVV. The most predominant of these include height, sex, and age [25, 26]. Studies have shown that individuals who smoke [27], suffer from cystic fibrosis [28], and women who are pregnant [29] also exhibit MVV values that deviate from height-, sex- and age-matched controls. Furthermore, a growing body of literature suggests that current reference equations for MVV and other PFTs fail to account for ethnic and socioeconomic disparities in addition to ignoring the trend of increasing racial diversity [30, 31]. Investigations have derived specific prediction equations based on ethnicity, such as in Brazilian [11], Chinese [13], Filipino [32], and African-American adolescent [12] populations, but all possess significant mathematical differences from one another. The recurring shortcoming of these equations is their lack of cohesion—no comprehensive equation exists that successfully incorporates all of the aforementioned variables. As a result, a number of studies have argued that MVV is best measured directly [19, 28, 33].

It is important to note that the results from this investigation possess similar limitations to those outlined above, including a homogenous participant cohort and the inability to account for differences in physical characteristics due to a small sample size. Further research ought to examine whether a self-selected *f*_R_ is as accurate and more efficient than a preselected range of *f*_R_ in a larger, more heterogeneous population. Future investigations should also explore other methods to standardize the assessment of MVV, for instance, an optimal test duration and the feasibility of a definitive reference equation. Until then, we advise that participants perform the maneuver preferably at a self-selected *f*_R_ or within a preselected range of 90–120 breaths·min^− 1^.

## Conclusion

Although not as prominent as in decades past, MVV remains a clinically relevant PFT whose outcomes are valuable in cardiopulmonary exercise testing and aid in the diagnosis of various neuromuscular, cardiovascular, and pulmonary diseases. This study demonstrates the shortcomings of the current lack of standardization in MVV testing and establishes data-driven recommendations for optimal *f*_R_. While classic literature has previously investigated this topic, the antiquity of these works warrants modern research. Furthermore, what these studies suggest as an optimal measurement technique has been overlooked or forgotten in the current guidelines. Our paper therefore contributes a new and much-needed focus on an evidence-based approach to selecting the optimal *f*_R_ for MVV measurement. We recommend that participants perform an MVV maneuver at a self-selected *f*_R_ as it maximizes the likelihood of an accurate measurement by optimizing a combination of slower deep breaths and faster shallower breaths, eliminates the necessity to synchronize breaths to a rhythm-keeping device, and more closely resembles the procedure to obtain ${\dot{V}}_{Emax}$. If an individual is unable to perform or performs the maneuver poorly at a self-selected *f*_R_, ventilating within a mandated *f*_R_ range of 110–120 breaths·min^− 1^ may also be acceptable.

## Abbreviations

ATS/ERS: American Thoracic Society/European Respiratory Society
FEV_1_: Forced expiratory volume in 1 s
*F*_R_: Breathing frequency
*f*_Rself_: Self-selected breathing frequency
*f*_RX_: Preselected breathing frequency of X breaths·min^−1^
MVV: Maximum voluntary ventilation
PETCO_2_: Partial pressure of end-tidal CO_2_
PFT: Pulmonary function test
SD: Standard deviation
SVC: Slow vital capacity
UCLA: University of California, Los Angeles
${\dot{V}}_{Emax}$: Maximal exercise ventilation
V_T_: Tidal volume
